# Multi-institutional assessment of HER2 immunohistochemistry in gastric and esophagogastric junction cancer

**DOI:** 10.1016/j.isci.2026.115635

**Published:** 2026-04-24

**Authors:** Xiu Zhu, Lan Yang, Yangqin Zheng, Danni Zhu, Nan Li, Yanxia Ying, Haiqiang Wang, Feng Wang, Xiaoxiao Yang, Xing Liu, Guanglin Liao, Yihua Wang, Menger Wang, Guangjun Jin, Xiaoyan Zhou, Xiaoxiao Wu, Jingjing Xu, Ding Wang, Zhenyuan Liu, Yunying Liu, Xiaozhen Yu, Fei Wu, Shujue Zhao, Hui Wang, Jieer Ying, Xiangdong Cheng, Qing Wei

**Affiliations:** 1 Department of Pathology, Zhejiang Cancer Hospital, Hangzhou, China; 2 The First People’s Hospital of Jiande, Hangzhou, China; 3 Department of Hematology, The Third Clinical Institute Affiliated to Wenzhou Medical University (Wenzhou People’s Hospital), Wenzhou, China; 4 Department of Gastric Surgery, Zhejiang Cancer Hospital, Hangzhou, China; 5 Wenzhou Medical University, Wenzhou, China; 6 The Fourth Affiliated Hospital of Harbin Medical University, Harbin, China; 7 Department of Pathology, XianJu People’s Hospital, Xianju, Zhejiang, China; 8 Shulan (Hangzhou) Hospital, Hangzhou, China; 9 The Second Affiliated Hospital of Zhejiang Chinese Medical University, Hangzhou, China; 10 Department of Medical Oncology, Zhejiang Cancer Hospital, Hangzhou, China

**Keywords:** Oncology, Pathology

## Abstract

The reliability of the traditional HER2 immunohistochemistry (IHC) scoring system for classifying low-expression cases remains uncertain, creating a barrier to effectively deploying new antibody-drug conjugates. To assess inter-observer agreement, 15 pathologists independently scored HER2 IHC in 460 gastric cancer specimens. We found the highest overall percent agreement (OPA) for HER2 3+ scores (0.17). The OPA for HER2 IHC 2+ cases was 0.11. However, consensus could not be reached for specimens scored as 0 or 1+. These findings suggest that conventional HER2 IHC scoring is relatively reliable for identifying patients with high HER2 expression but reveals significant inconsistency in classifying HER2-low versus HER2-zero cases. This highlights a critical limitation of the current system for guiding modern therapies and underscores the necessity for revised, precise scoring methodologies.

## Highlights

• HER2 IHC scoring has high inter-observer variability, particularly between HER2 0 and 1+
• Defining a combined “HER2-low” category (1+/2+) improves scoring agreement
• HER2 scoring consistency is strongly influenced by pathologist experience

## Graphical abstract

**Figure:**
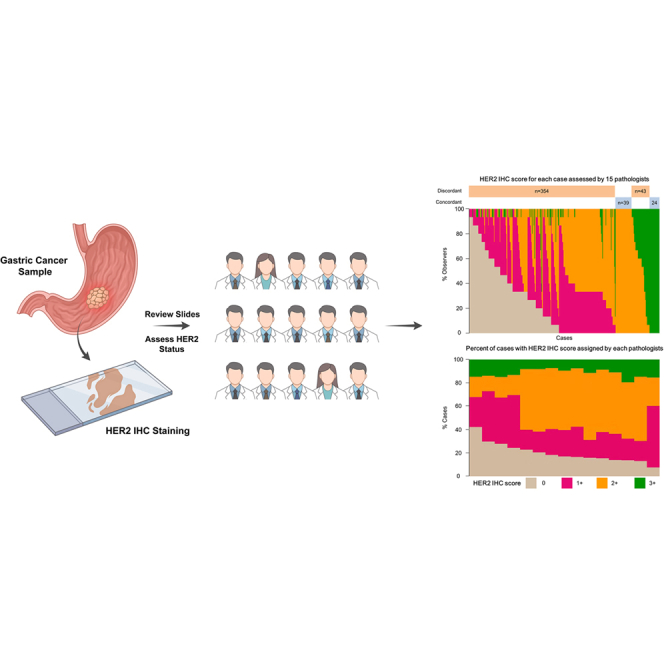

## Introduction

Antibody-drug conjugates (ADCs) represent a class of therapeutics that selectively and efficiently deliver cytotoxic agents to tumor tissues, exhibiting substantial antitumor efficacy within a broad therapeutic spectrum.^1^ Described as “magic bullets,”^2^ ADCs have become a focal point in oncology, particularly in the clinical research of HER2-targeted therapies. Human epidermal growth factor receptor 2 (HER2), recognized as a pivotal therapeutic target, has garnered significant attention in ADC drug development.^3^ Notably, trastuzumab deruxtecan (T-DXd, DS-8201a), a third-generation ADC, has significantly transformed the treatment landscape for HER2-expressing tumors.^4^

Traditionally, HER2-targeted therapies have focused on patients with HER2-positive breast or gastric cancer/gastroesophageal junction cancers (GC/GEJC), as the benefits were primarily observed in those with amplified or overexpressed HER2, exemplified by trastuzumab’s efficacy.^5^^,^^6^^,^^7^ Clinical practices have thus been oriented toward detecting high HER2 expression levels. Previous research has established a strong linear correlation between HER2 expression levels and the efficacy of targeted therapies.^8^^,^^9^ The Ventana 4B5 assay, a widely used companion diagnostic test, performs optimally in tumors with over 100,000 HER2 molecules per cell. Despite this, the American Society of Clinical Oncology(ASCO)/College of American Pathologists (CAP) guidelines mandate fluorescence in situ hybridization (FISH) testing for immunohistochemistry (IHC) 2+ cases to ensure HER2-amplified tumors are not overlooked.^10^^,^^11^ With the advent of novel ADCs and an expanded understanding of bystander effects and other mechanisms, attention has shifted to HER2 low-expression populations.^1^^,^^12^^,^^13^ T-DXd has a potent bystander effect due to a highly membrane-permeable payload and is beneficial in treating tumors with HER2 heterogeneity or with low HER2 expression.^1^^,^^14^ Studies like DB06 and DAISY in breast cancer have demonstrated therapeutic efficacy in tumors initially classified as HER2 0, which suggests potential benefits for these patients from ADC therapy.^15^^,^^16^ Subsequent analyses have revealed that some HER2 0 tumors may be more accurately classified as HER2 1+,^17^ and HER2 IHC 0 versus low status appears unstable across patient samples.^18^ Recent clinical trials for T-DXd have sought to delineate the low HER2-expressing subgroup as 1+ or 2+ cases without gene amplification, adhering to the 2018 ASCO/CAP guidelines originally designed for detecting amplified HER2 expression. This evolution raises questions about the adequacy of legacy HER2 assays, initially approved by the FDA for historical drugs, in their application to ADCs.

Historically, the distinction between HER2 0 and 1+ (or “low expressing”) cases did not necessitate precise, reproducible concordance, as trastuzumab was not utilized in HER2-negative patients.^19^ However, the ability to distinguish “true negative” from HER2-low cases is now clinically significant for the administration of emerging therapies such as T-DXd. The 2023 ASCO/CAP guideline update reflects this clinical relevance.^11^ The groundbreaking results of the DESTINY-Breast04 (DB04) trial demonstrated that T-DXd significantly outperforms traditional chemotherapy in patients with HER2-low metastatic breast cancer, establishing the first ADC effective against tumors previously classified as HER2-negative.^12^ This has prompted rapid expansion of low HER2 expression research across multiple solid tumor types, including GC/GEJC. However, a major challenge remains in the conventional pathological interpretation of HER2 status: the distinction between IHC 0 and 1+ is highly subjective and poorly reproducible, often leading to misclassification of eligible patients as HER2-negative. This diagnostic ambiguity risks excluding patients who could benefit from novel ADC therapies. Recent studies have highlighted considerable interrater variability and poor reproducibility in differentiating low versus “true negative” (IHC 0) HER2 cases in breast cancer.^20^^,^^21^^,^^22^^,^^23^ Lambein et al.^22^ reported up to 85% disagreement in HER2 0 IHC scores using the Ventana 4B5 assay between local and central laboratories. A retrospective analysis in breast cancer involving five specialized pathologists revealed that discordance was predominantly driven by 0 vs. 1+ cases, accounting for 43% of all discordant instances and 15% of total cases.^23^ Despite these findings, the limited number of pathologists and cases in these studies restricts definitive conclusions on interrater reliability. Furthermore, conventional methods for assessing interrater reliability among a small cohort may not generalize effectively to the broader population of pathologists. There remains no established methodology for evaluating concordance among a large number of observers, nor a statistical standard for determining the minimum number of observers required to reflect real-world pathologist performance.

In GC/GEJC, studies such as Destiny-GC01 have indicated that patients with low HER2 expression may also derive therapeutic benefit.^24^ Pan-tumor studies suggest a certain response rate among patients with GC/GEJC with low HER2 expression.^11^ However, large-scale studies in this area are limited, and GC/GEJC’s molecular and pathological heterogeneity poses challenges for systematic review and analysis.^25^^,^^26^ The assessment of HER2 expression in GC/GEJC is particularly challenging, casting doubt on the established cut-off points in current guidelines.^27^^,^^28^

As ADC development progresses rapidly, the necessity for accurate target detection has become increasingly apparent. The misassignment of patients in treatment allocation due to inadequate HER2 assays could undermine therapeutic efficacy, as highlighted in breast cancer studies.^29^ Given HER2’s pivotal role in GC/GEJC therapy, greater precision in detection and interpretation is imperative. Despite the abundance of literature on breast cancer, large-scale pathological studies on GC/GEJC remain scarce.

To address these gaps, we conducted a multi-institutional study evaluating the interrater reliability of HER2 IHC scoring in 460 GC/GEJC cases, involving 15 pathologists specializing in GC/GEJC.

## Results

### Cohort characteristics and HER2 IHC assessment workflow

**Figure 1:**
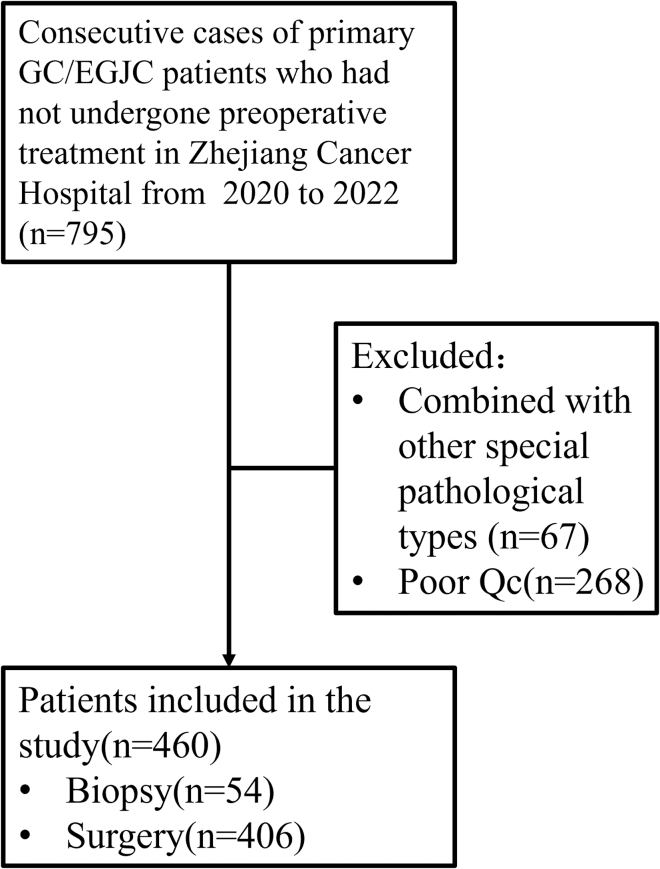
Flow chart Case screening flow chart showing the selection process of patients and the causes for exclusion. GC/EGJC: gastric and esophagogastric junction cancer; Qc, quality control.

**Figure 2:**
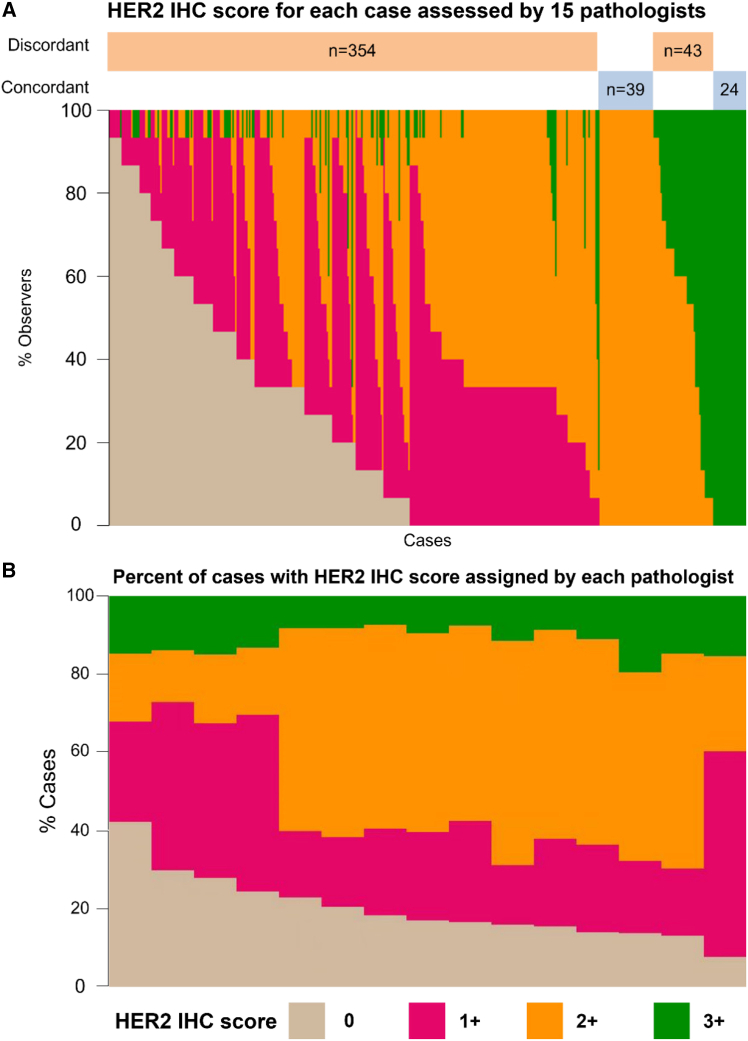
HER2 IHC scores for 460 cases read by 15 pathologists (A) Each bar represents an individual case on the *x* axis, displayed as the proportion (%) of observers assigning HER2 IHC scores of 0, 1+, 2+, or 3+. Concordant (100% agreement) and discordant cases are indicated by bars above the plot. (B) Distribution of HER2 IHC scores assigned by each of the 15 pathologists, shown as the percentage of cases in each category. This figure presents the raw distribution of pathologists’ scores for each individual case (*n* = 460). Data are presented as the actual proportion (percentage) of the 15 observers assigning each HER2 IHC category for every sample. HER2, human epidermal growth factor 2; IHC, immunohistochemistry.

In this study, 795 consecutive cases of primary gastric and esophagogastric junction cancer at Zhejiang Cancer Hospital were retrospectively reviewed. Two pathologists reviewed the slides and excluded cases that failed to meet the predefined quality control criteria. Finally, this study analyzed clinicopathological data from 460 patients with GC/GEJC (Figure 1). The clinical and pathological data of the patients are provided in the supplemental table (Table S1). This study did not assess the association between patient sex and HER2 scores due to its design focus on inter-observer agreement. To evaluate the interrater reliability of HER2 IHC scoring, this study involved 15 pathologists from various institutions to assess HER2 IHC on biopsy or surgical specimens obtained from patients who had not undergone preoperative treatment. Figure 2 shows stacked bar plots of HER2 IHC scores assigned per case (Figure 2A) and by each pathologist (Figure 2B).

### High discordance in HER2 IHC scoring is concentrated between 0 and 1+ categories

Among the 460 cases, 397 demonstrated scoring discrepancies across the 15 pathologists, with an overall percent agreement (OPA) of 0.14. Comparing pathologists’ concordance using a 4-category score (0, 1+, 2+, 3+) and a 3-category score (0, low, 3+), we found that the 3-category scoring system yielded a higher OPA (0.14 for the 4-category versus 0.38 for the 3-category) (Table 1). This is also illustrated in Figure 2A, where combining 1+ and 2+ categories removes 114 discordant cases. Similarly, Fleiss’ kappa shows an increase when using a 3-category scoring system compared to a 4-category system for HER2 IHC assessment (Fleiss’ kappa of 0.41 for the 4-category vs. 0.50 for the 3-category scores). This finding suggests that there is substantial discordance in distinguishing between 1+ and 2+ cases, merging 1+ and 2+ into one category may improve concordance.

**Table 1:** Summary of interrater reliability metrics for different HER2 IHC groups among 15 pathologists in 460 cases of gastric cancer

HER2 IHC group	Overall percent agreement(95%)	Fleiss’kappa(95%)	ICC(95%)
4-category score (0, 1+, 2+, 3+)	0.14 (0.11–0.17)	0.41(0.41,0.41)	0.65(0.62–0.68)
3-category score (0, low^a^, 3+)	0.38 (0.34–0.43)	0.50(0.50,0.50)	0.65(0.61–0.68)

**Including cases with only this score assigned by at least 1 pathologist**			

0 only	0.00 (0.00–0.02)	0.22(0.22–0.22)	0.27(0.22–0.32)
1+ only	0.00 (0.00–0.01)	0.27(0.27–0.27)	0.44(0.39–0.50)
2+ only	0.11 (0.08–0.15)	0.30(0.30–0.30)	0.45(0.40–0.51)
3+ only	0.17 (0.11–0.23)	0.44(0.39–0.44)	0.65(0.59–0.71)
low only	0.35 (0.31–0.40)	0.36(0.36–0.36)	0.55(0.51–0.60)
0 vs. not 0	0.53 (0.48–0.57)	0.43(0.43–0.43)	0.43(0.39–0.47)
1+ vs. not 1+	0.23 (0.19–0.27)	0.23(0.23–0.23)	0.23(0.20–0.27)
2+ vs. not 2+	0.30 (0.26–0.35)	0.44(0.44–0.44)	0.45(0.39–0.50)
low vs. not low	0.62 (0.57–0.66)	0.46(0.46–0.46)	0.46(0.43–0.50)
3+ vs. not 3+	0.74(0.69–0.78)	0.67(0.67–0.67)	0.67(0.64–0.70)
<2+ vs. ≥ 2+	0.77 (0.73–0.81)	0.51(0.51–0.51)	0.51(0.46–0.56)

HER2, human epidermal growth factor 2; IHC, immunohistochemistry; ICC, intraclass correlation coefficient; 95% CI, 95% confidence interval.

a The low category is the result of combining the 1+ and 2+ categories.

Discordance was observed across all scoring cut points, with the greatest number of discrepancies occurring in scores of 0 and 1+. Of the 460 cases, 217 received a score of 0 from at least one pathologist, yet none of these cases achieved full concordance (OPA = 0.00) (Figure 2A; Tables 1, 2 and 3). Similarly, 337 received a score of 1 from at least one pathologist, and none of these cases achieved full concordance (OPA = 0.00) (Figure 2A; Tables 1, 2 and 3). Previous studies indicate that the primary discordance in HER2 IHC scoring arises from discordance between scores of 0 and 1+.^23^ With concerning rates of discordance (85%) reported between local and central HER2 0 assessments.^22^ Figures S1A and S1B show the OPA Observers Needed to Evaluate a Subjective Test (ONEST) plots when cases were scored as 0 and 1+, respectively, by at least 1 of the 15 pathologists. The OPA for the cases that were scored as 0 plateaus at 0.00, reflecting 100% disagreement on cases which at least one pathologist rated them as 0. Most discordance for 0 cases occurred between scores of 0 and 1+ (1136/3255 ratings, 200/217 cases read as 1+ by another pathologist), and to a lesser extent between 0 and 2+ scores (661/3255 ratings, 140/217 cases) (Tables 2 and 3). The OPA for HER2 IHC 1+ cases was also 0.00, as there was no case among the 337 scored as 1+ by at least one pathologist that all 15 pathologists agreed upon (Figure 2A; Tables 1 and 3). The OPA for HER2 IHC 2+ cases was 0.11, as there were 39 cases among the 359 scored as 2+ by at least one pathologist for which all 15 pathologists agreed (Figure 2A; Tables 1 and 3). As for cases that were scored as 3+, 145 cases were scored as 3+ by at least one pathologist, with 24 achieving full concordance among all raters (OPA = 0.17) (Figure 2A; Tables 1 and 3). This also indicates that even with the critical pathological assessment of HER2 IHC 3+, there remains a high level of inconsistency among raters. However, it is still observed that there is the least significant scoring variability in HER2 IHC 3+, with pathologists classifying 7.39%–19.57% of cases as IHC 3+, while the variability in other groups was substantially higher (Figure 2B). The OPA for the cases that were scored as 3+ plateaus with approximately 12 raters (Figure S1C), which reveals that 12 pathologists are necessary to obtain reliable concordance estimates.

**Table 2:** Amount of HER2 IHC ratings for each subset of cases among 15 pathologists and 460 cases of gastric cancer

HER2 IHC group	0 counts	1+ counts	2+ counts	3+ counts	Total Ratings
0 only	1374	1136	661	84	3255
1+ only	1282	1873	1753	147	5055
2+ only	652	1469	2829	435	5385
3+ only	338	409	604	824	2175

HER2, human epidermal growth factor 2; IHC, immunohistochemistry. Each subset is defined by including cases with the score assigned by at least one pathologist.

**Table 3:** Number of HER2 IHC cases scored by at least one pathologist for each subset of cases among 15 pathologists and 460 cases of gastric cancer

HER2 IHC group	0 counts	1+ counts	2+ counts	3+ counts	Total Cases
0 only	217	200	140	55	217
1+ only	200	337	261	74	337
2+ only	140	261	359	92	359
3+ only	55	74	92	145	145

HER2, human epidermal growth factor 2; IHC, immunohistochemistry. Each subset is defined by including cases with the score assigned by at least one pathologist.

### Clinical decision thresholds (0 vs. not 0, 2 vs. not 2) show suboptimal agreement

**Figure 3:**
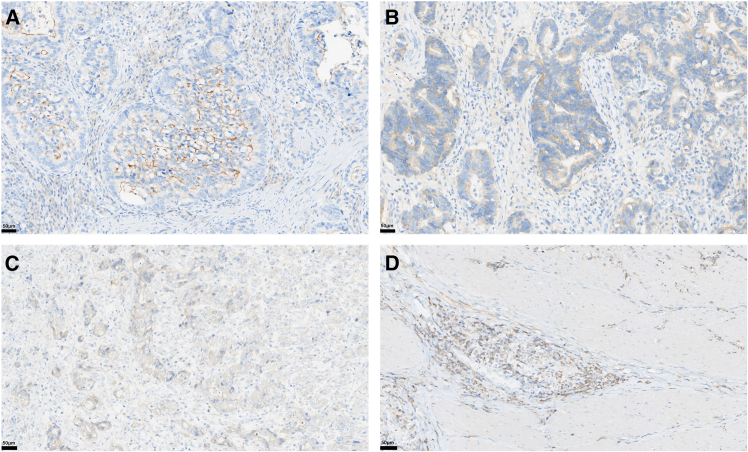
Representative cases that were misinterpreted HER2 IHC results that were truly score 0 were misclassified as score 1: (A) The luminal membrane positivity in tumor cells should not be counted. (B) The proportion of positive cells did not reach the 10% threshold required for a 1+ score. HER2 IHC results that were truly score 1 were misclassified as score 0: (C–D) The proportion of positive cells reached the 10% threshold.

Since the recognition of lower levels of HER2 as a therapeutic target in multiple clinical trials, distinguishing between cases scored as 0 vs. not 0 has become a crucial clinical decision threshold for prescribing HER2-low therapies. Compared to the classification of 3 vs. not 3, the OPA for 0 vs. not 0 was significantly lower (0.53 for 0 vs. not 0 compared to 0.74 for 3 vs. not 3). This highlights the challenge in identifying which patients could potentially receive new-generation ADCs like T-DXd, unlike the relatively clearer criteria for trastuzumab administration. These findings are consistent with previous studies showing that pathologists struggle to reliably identify HER2 IHC 0 cases, with up to 0.47 disagreement on whether cases should be scored as IHC 0 or not 0. The misinterpretation of HER2 IHC 0 scores can be attributed to a variety of factors. Figure 3 shows representative cases that were misinterpreted.

Trastuzumab is prescribed for HER2-amplified cases defined as 3+ or 2+ with ISH positivity. Therefore, establishing the threshold between 2 vs. not 2 is essential and has been a central focus of previous HER2 research, as HER2-positive status for HER2 IHC 2+ requires FISH testing for confirmation. The OPA for 2 vs. not 2 was 0.30 (0.26–0.35), plateauing with approximately seven raters (Figure S1E). This indicates that 70% of pathologists did not reach a consensus on which patients require complementary FISH testing, underscoring the variability and uncertainty in current diagnostic practices.

### Pathologist experience and specimen type influence scoring consistency

The diagnostic experience and subspecialty expertise of pathologists may significantly influence HER2 IHC scoring consistency in GC/GEJC. In this study, the 15 pathologists were stratified by experience: 8 had ≥5 years of specialized experience in interpreting gastrointestinal (GI) tumor slides, including HER2 IHC scoring in routine diagnostic practice, while 7 had <5 years. Cases reviewed by pathologists with ≥5 years of experience demonstrated a 0.22 higher OPA (OPA = 0.40 vs. 0.18) (Table S2), supporting the ASCO/CAP recommendations that HER2 assessment should be performed by experienced GI pathologists.

Given the specimen composition (54 biopsies vs. 406 surgical specimens), we also conducted an additional analysis comparing pathologists' interpretation accuracy between different specimen types(Table S3). The OPA for 0 vs. not 0 was significantly lower (0.35 for biopsies compared to 0.55 for surgical specimens), indicating that sufficient tissue is warranted to identify HER2 expressions.

## Discussion

This study assessed the discrepancies among pathologists in interpreting HER2 IHC scores from pathological specimens of 460 patients with GC/GEJC. The findings align with prior studies examining HER2 pathological diagnostics in gastric and breast cancers.^30^^,^^31^^,^^32^ However, previous research involved fewer pathological specimens and a smaller cohort of pathologists. Thus, this study represents the largest multi-institutional investigation to date on HER2 IHC scoring interpretation in GC/GEJC.

The greatest discrepancies in this study occurred in HER2 0 and HER2 1+ scores, mirroring observations in breast cancer.^29^^,^^33^ Notably, breast cancer studies have demonstrated that patients with HER2 1+ can benefit from T-DXd treatment.^34^ Furthermore, more than half of breast cancer cases classified as HER2 IHC 0 exhibited significant HER2 expression when assessed using quantitative immunofluorescence.^17^ This underscores the necessity to re-evaluate whether patients with GC/GEJC classified as HER2 0 are truly HER2-negative. Historically, distinguishing between HER2 0 and HER2 1+ was not stringently required in clinical practice. This study highlights important considerations regarding HER2 assessment in GC/GEJC, particularly as current clinical trials for ADC therapies increasingly include patients across the HER2 expression spectrum rather than being limited to HER2-positive cases. Our findings underscore the need to better characterize real-world HER2 interpretation practices.

In addition to interpretation variability, digital pathology itself presents technical challenges. Virtual slides may compromise the discernibility of subtle staining gradients in weakly positive (1+) cases, potentially affecting scoring accuracy. These findings highlight two critical needs for future research: (1) implementing complementary physical slide reviews to enhance diagnostic precision in borderline cases, and (2) developing more rigorous standardization guidelines for HER2 0 versus 1+ differentiation in digital pathology. Such advancements become increasingly crucial as eligibility criteria of clinical trials expand to include patients with low HER2 expression in current practice.

Not surprisingly, the inconsistencies among pathologists interpreting HER2 scores in GC/GEJC are significantly greater than those observed in breast cancer. We believe these discrepancies do not stem from a single factor but rather result from a combination of biological, histological, and technical factors. Compared with breast cancer, GC/GEJC often exhibit more pronounced spatial heterogeneity—HER2 expression may demonstrate a “mosaic” distribution within the same tissue sample, with HER2-positive and HER2-negative regions closely intermingled. In breast cancer, HER2 positivity is typically associated with certain specific subtypes (such as ductal carcinoma) and relatively consistent morphology. In contrast, GC/GEJC display greater diversity in histological subtypes (e.g., intestinal, diffuse, and mixed types), contributing to more complex interpretation. HER2 staining in GC/GEJC is often less “typical” and “strongly positive” compared to breast cancer. While breast cancer cases with 3+ scores usually show complete, intense circumferential membrane staining that is visually striking, positive staining in GC/GEJC tends to be weaker and more discontinuous. Procedural artifacts from endoscopic biopsy handling (such as pinching and crush injuries) and suboptimal fixation processes (e.g., delayed fixation or inadequate penetrance of fixative) may further compromise tissue integrity and staining quality. Moreover, HER2 testing in GC/GEJC has a shorter history of clinical application, and pathologists have relatively less experience in interpreting its diverse and often atypical staining patterns, which naturally leads to higher interobserver variability. To address these challenges, we recommend implementing a multi-tiered strategy, including enhanced training and standardization protocols, mandatory dual-pathologist review, and the integration of auxiliary technologies such as digital pathology and artificial intelligence (AI).

This discrepancy may partially explain why ADC treatments for GC/GEJC have not achieved the same efficacy as in breast cancer. The variability in HER2 scoring among pathologists suggests potential selection bias in patient enrollment. Although the conventional HER2 IHC assay combined with ISH remains the companion diagnostic for ERBB2-amplified therapies, such as trastuzumab,^35^ the high discordance and low interrater reliability in scoring IHC 0, 1+, and 2+ cases indicate potential issues when applying this assay to emerging HER2-low therapies. The efficacy of T-DXd in HER2-low patients in DB04 has already surpassed that of traditional chemotherapy.^12^ Similar exploration of low HER2 expression is underway in various other tumor types (pan-tumor).^36^ However, in conventional interpretation, the distinction between HER2 IHC 0 and 1+ is ambiguous, which easily leads to misclassification of patients as HER2-negative and consequently causes them to miss the opportunity for ADC-based therapy. Furthermore, in breast cancer, T-DXd has outperformed trastuzumab even in first-line treatment for HER2-high patients,^37^ and similar studies are being conducted in GC/GEJC.^38^ This underscores that accurate HER2 interpretation is critical—whether for classifying high or low expression—and significantly influences anti-HER2 treatment decisions. Thus, it highlights that the current scoring system, tailored for traditional treatments, may not adequately meet the demands of novel therapeutic advancements.

Consistent with other studies employing alternative methodologies, the high level of discordance in HER2 IHC 0 vs. not 0 scoring reported in this study suggests that current IHC detection methods are suboptimal for identifying low levels of HER2 expression. This could lead to false-negative or false-positive results near the IHC 0/IHC 1+ threshold, potentially resulting in incorrect treatment recommendations as HER2-low therapies develop. It is essential that pathology tests achieve high inter- and intra-observer concordance to ensure equitable treatment outcomes. Moreover, concordance should be linked to clinical outcomes. There is a pressing need to redefine the subgroups of GC/GEJC with a more precise approach to determine if patients are “targetable” and “distinguishable” for subsequent treatments.

### Limitations of the study

This study has several limitations that warrant consideration. First, while employing conventional pathologist evaluation methods, we did not incorporate AI-assisted quantitative analysis (such as HER2 membrane staining AI scoring algorithms) or automated image analysis software. During the training phase, AI models integrate diverse sample data and real-world scenarios—including variables like staining intensity, section thickness, and background noise—thereby achieving strong generalization capability. A key advantage of AI lies in its visual image analysis: it can identify membrane staining characteristics of individual tumor cells, such as continuity of staining and whether intensity exceeds a threshold, while maintaining objective judgment even in the presence of uneven staining or background interference. This technology enables visual and quantitative discrimination of subtle differences between IHC 0 and 1+, significantly enhancing the ability to identify HER2 ″ultra-low expression” cases.^39^ Moreover, AI systems provide transparent and traceable outputs, aligning with the principles of interpretability emphasized in the ASCO/CAP 2023 guidelines. With AI assistance, pathologists have demonstrated significantly improved diagnostic accuracy and inter-observer agreement, particularly showing higher reliability in interpreting faint staining. Although such technologies are emerging rapidly and hold promise for reducing human bias and enhancing reproducibility, future research should further explore the complementary role of AI in traditional pathological assessment. It is important to emphasize that physicians retain final decision-making authority after considering AI-generated suggestions—this human-AI collaborative model represents the direction of future development.^40^ Second, despite all HER2 scoring being performed by experienced pathologists with bias mitigation through double-blind review and consensus meetings, subtle interobserver variations in staining intensity interpretation may persist among pathologists of different experience levels. More rigorous standardized training (e.g., specialized digital pathology HER2 interpretation courses) or multicenter cross-validation could further improve scoring consistency. Additionally, the 15 participating pathologists were not informed of the study objectives during sample scoring. While prior training on low-score assessments might enhance consistency,^35^ disclosing the study purpose could have influenced their routine scoring practices—a methodological choice designed to better reflect real-world clinical scenarios. Finally, the study lacked quantitative molecular analysis of HER2 expression in core biopsy samples, which, while mirroring current clinical practice where precise HER2 quantification methods are often unavailable, highlights an important diagnostic limitation. Collectively, while our study design effectively simulated actual clinical diagnostic processes, these limitations underscore existing challenges in HER2 interpretation and point to potential avenues for future improvement in pathological assessment methodologies.

## Resource availability

### Lead contact

Further information and requests for resources should be directed to and will be fulfilled by the lead contact, Qing Wei (weiqingmd@163.com).

### Materials availability

This study did not generate new unique reagents.

### Data and code availability

• Data: The original whole-slide images (WSIs) used for HER2 scoring are protected patient data and cannot be publicly shared due to patient privacy and confidentiality regulations. However, the complete, de-identified dataset underlying all statistical analyses and conclusions in this study has been deposited at Zenodo and is publicly available. This dataset includes the HER2 IHC scores (0, 1+, 2+, 3+) assigned by each of the 15 pathologists to each of the 460 GC cases. The data can be accessed via Zenodo: https://doi.org/10.5281/zenodo.18357643. A direct link to the dataset is: https://doi.org/10.5281/zenodo.18357643
• Code: This study did not generate original code.
• Additional Resources: Any additional information required to reanalyze the data reported in this article is available from the lead contact upon request.

## Acknowledgments

This study was supported by the Zheijiang Province Traditional Chinese Medicine Science and Technology Plan Proiect (grant no. 2024ZL301 [Q.W.]); the 10.13039/501100001809National Natural Science Foundation of China (grant no. 82303963 [Q.W.]); CSCO young funding (grant no. Y-Young 2023-0193 [Q.W.]); and the CSCO HER2-Related Solid Tumor Research Fund Project (grant no. Y-2022HER2AZQN-0353 [Q.W.]). The authors thank Huiliang Huang for data interpretation.

## Author contributions

Conceptualization, X.Z.(Xiu Zhu), L.Y., Y.Z., D.Z., J.Y., X.C., and Q.W.; data curation, X.Z.(Xiu Zhu), L.Y., Y.Z., D.Z., N.L., Y.Y., H.W., F.W.(Feng Wang), X.Y.(Xiaox Yang), X.L., G.L., Y.W., M.W., X.Z.(Xiaoy Zhou), X.W., J.X., D.W., Z.L., Y.L., X.Y.(Xiaoz Yu), F.W.(Fei Wu), S.Z., and H.W.; formal analysis, X.Z.(Xiu Zhu), L.Y., Y.Z., D.Z. , G.J., and Q.W.; funding acquisition, Q.W.; investigation, X.Z.(Xiu Zhu), L.Y., Y.Z., D.Z., N.L., Y.Y., H.W., F.W.(Feng Wang), X.Y.(Xiaox Yang), X.L., G.L., Y.W., M.W., G.J., X.Z.(Xiaoy Zhou), X.W., J.X., D.W., Z.L., Y.L., X.Y.(Xiaoz Yu), F.W.(Fei Wu), S.Z., and H.W.; project administration, J.Y., X.C., and Q.W.; resources, J.Y., X.C., and Q.W.; supervision, J.Y., X.C., and Q.W.; visualization, X.Z.(Xiu Zhu), L.Y., Y.Z., and D.Z.; writing – original draft, D.Z. and Q.W.; writing – review and editing, D.Z. and Q.W.

## Declaration of interests

The authors declare no competing interests.

## STAR★Methods

### Key resources table

**Table:** 

REAGENT or RESOURCE	SOURCE	IDENTIFIER
**Deposited data**		

De-identified HER2 IHC scoring dataset	This paper	https://doi.org/10.5281/zenodo.18357643

**Antibodies**		

Anti-HER2/ERBB2 antibody (clone 4B5)	Ventana Medical Systems (Roche)	RRID: AB_2921204

**Software and algorithms**		

IBM SPSS Statistics for Windows, version 26.0	IBM SPSS	https://www.ibm.com/cn-zh/products/spss
R version 4.1.0	The R Foundation	https://www.r-project.org/
ONEST R package	Han et al., 2022	https://doi.org/10.1002/sim.9282

### Experimental model and study participant details

#### Human participants/specimens

This study utilized archived, fully de-identified tissue specimens from patients diagnosed with gastric or esophagogastric junction adenocarcinoma.

#### Sample size

A total of 460 cases were included in the final analysis after quality control screening from an initial 795 consecutive cases.

#### Sex distribution and analysis

The cohort included male and female patients, with the distribution provided in Table S1. As the primary aim of this study was to assess inter-observer agreement in HER2 scoring—a metric focused on the consistency of pathologists’ interpretations rather than patient outcomes—the potential association between patient sex and scoring results was not a predefined analytical endpoint. Therefore, the influence of patient sex on HER2 IHC scoring concordance was not statistically evaluated in this analysis.

#### Ethics approval

This study was a retrospective observational study that analyzed only previously collected and fully de-identified medical records. Given the nature of the study—no patient intervention, minimal risk, and the impracticability of obtaining current informed consent—ethical approval was obtained from the Ethics Review Committee of Zhejiang Cancer Hospital (approval No. IRB-2025-62(IIT)), with a waiver of the requirement for informed consent. This study was conducted in strict accordance with the Declaration of Helsinki and relevant Chinese ethical regulations, and all patient data were kept confidential.

#### Specimen details

Specimens consisted of surgical resection samples (*n* = 406) and endoscopic biopsy samples (*n* = 54), collected at Zhejiang Cancer Hospital between 2020 and 2022.

#### Pathologist raters

Fifteen board-certified pathologists from multiple institutions participated as observers. Their experience level was stratified for analysis: 8 had ≥5 years of specialized experience in gastrointestinal pathology, and 7 had <5 years.

### Method details

#### Tissue processing and staining

All specimens were fixed in 10% neutral buffered formalin under standardized conditions. Surgical specimens all obtained from radical resection of fresh tissues and fixed in 10% neutral buffered formalin within 30 min after excision and endoscopic biopsy specimens directly immersed in the same fixative after endoscopic forceps collection. All specimens were subjected to strictly controlled fixation times of 24–48 h (24 h for biopsy specimens, ≤48 h for surgical specimens), with the entire fixation and storage process maintained at room temperature (20°C–25°C).

The same technical team performed dehydration, embedding, and sectioning (4 μm thickness) using an automated platform (Ventana Benchmark XT) with FDA-approved primary antibody (clone 4B4). Each batch included positive and negative controls.

#### Quality control (QC) for slide inclusion

All archived H&E-stained slides and HER2 immunohistochemical slides were reviewed to ensure good staining quality, intact slides, and sufficient tissue for evaluation. A standard quality control (QC) process typically involves the following steps.

1) Initial screening (macroscopic examination): check slide integrity, labeling, and for obvious issues such as folds or mounting errors.
2) Microscopic Examination at Low Magnification (e.g., 4x, 10x objective): Assess overall tissue integrity, general morphology, approximate quantity and distribution of tumor cells, and presence of significant artifacts.
3) Microscopic Examination at High Magnification (e.g., 20x, 40x objective): Evaluate cellular morphology, staining quality (nuclear-cytoplasmic contrast, specificity of IHC staining, background), and fixation effectiveness.
4) Control Validation: specifically review positive and negative controls for IHC staining to ensure they meet expected results.
5) Final Assessment: based on the above criteria, determine whether the slide passes quality control.

#### Digital slide distribution and scoring

The slides were digitally scanned at 20× optical magnification using the IBL500 whole slide scanning system (Guangzhou Libo Medical Technology Co., Ltd.). These digital slides were then distributed to 15 board-certified pathologists, the majority of whom had over five years of experience, for HER2 scoring (0, 1+, 2+, or 3+)， according to current ASCO/CAP guidelines.^11^

#### ONEST analysis

The Observers Needed to Evaluate a Subjective Test (ONEST) method was employed to model the convergence of Overall Percent Agreement (OPA) with increasing numbers of hypothetical raters. The analysis was performed using R version 4.1.0.43. A detailed description of this method is provided in supplementary methods.

### Quantification and statistical analysis

#### Statistical software

Analyses were performed using IBM SPSS Statistics (version 26.0) and R (version 4.1.0).

#### Inter-rater reliability metrics

OPA quantifies the overall consistency in ratings, whereas Fleiss' kappa assesses the degree of agreement exceeding chance, thereby ensuring a rigorous analysis of inter-rater reliability. ICC was calculated using a two-way random-effects model, with absolute agreement as the relationship type and single rater as the unit of measurement.

#### ONEST analysis

The Observers Needed to Evaluate a Subjective Test (ONEST) package44 was employed to model and visualize the variation in OPA utilizing R version 4.1.0.43. A detailed description of this method is provided in the supplementary methods.

#### Definition of n and data presentation

In this study, “n” represents the number of independent gastric cancer cases (*n* = 460). The sample size n represents 460 gastric cancer cases. All statistical details, including exact *n* values and test definitions, are provided in the figure legends and Tables. Descriptive data are presented in the figures and tables. The figure present descriptive statistics.

#### Specification of statistical tests and asterisks

The specific statistical tests used (OPA, Fleiss’ kappa, ICC, ONEST) are named in the main text.

#### Figures and statistical reporting

Among the main figures, Figure 2 presents descriptive statistics (the distribution of pathologists' scores) and does not involve hypothesis testing with associated *p*-values; therefore, no asterisks denoting statistical significance are used in this figure. Figure 1 is a flowchart, and Figure 3 presents representative micrographs; neither contains statistical data representations requiring such annotation.

#### Location of statistical details

All detailed statistical results, including exact OPA values, kappa coefficients, ICC values, and plateau points from ONEST analysis, are reported in the main Results text, corresponding figure legends, and supplementary tables.
