# Supplementary material for: Multi-institutional assessment of HER2 immunohistochemistry in gastric and esophagogastric junction cancer
Source: iScience. 2026 Apr 24;29(5):115635. doi: 10.1016/j.isci.2026.115635 (PMC13127390; doi:10.1016/j.isci.2026.115635)
Supplement: Document S1. Figure S1 and Tables S1–S3 and supplementary methods [file mmc1.pdf]

iScience, Volume *29*

Supplemental information

Multi-institutional assessment of HER2 immunohistochemistry in gastric and esophagogastric junction cancer

Xiu Zhu, Lan Yang, Yangqin Zheng, Danni Zhu, Nan Li, Yanxia Ying, Haiqiang Wang, Feng Wang, Xiaoxiao Yang, Xing Liu, Guanglin Liao, Yihua Wang, Menger Wang, Guangjun Jin, Xiaoyan Zhou, Xiaoxiao Wu, Jingjing Xu, Ding Wang, Zhenyuan Liu, Yunying Liu, Xiaozhen Yu, Fei Wu, Shujue Zhao, Hui Wang, Jieer Ying, Xiangdong Cheng, and Qing Wei

Supplemental information

Multi-Institutional Assessment of HER2 Immunohistochemistry in Gastric and Esophagogastric Junction Cancer

Xiu Zhu[1,11], Lan Yang[2,11], Yangqin Zheng[3,11], Danni Zhu[4,5,11], Nan Li[6] , Yanxia Ying[7], Haiqiang Wang[7], Feng Wang[7] ,Xiaoxiao Yang[7], Xing Liu[8], Guanglin Liao[1], Yihua Wang[1], Menger Wang[1], Guangjun Jin[9], Xiaoyan Zhou[1], Xiaoxiao Wu[1], Jingjing Xu[1], Ding Wang[1], Zhenyuan Liu[1], Yunying Liu[1], Xiaozhen Yu[1], Fei Wu[1], Shujue Zhao[1], Hui Wang[1], Jieer Ying[10], Xiangdong Cheng[4], Qing Wei[10,12]

Supplementary methods: Detailed methodology for the ONEST concordance analysis.

A hundred curves (of 100 combinations for each group size) were generated for plotting, and 1000 curves were used to estimate the mean and 95% confidence interval of the ONEST plot. The resulting ONEST plots descend and can reach a nonzero plateau that can be validated by estimating the parameters of the statistical model described in the article by Han et al.[1] The ONEST model can also be used to estimate the number of raters needed to reach the plateau by calculating when the OPA difference between successive groups becomes clinically insignificant (ie, <0.5%). This approach is designed not only to estimate the number of observers required for evaluating a subjective test but also to forecast the performance of the test or biomarker in real-world settings involving thousands of pathologists. The identification of a plateau suggests that the metric is likely to remain consistent, even as the number of raters continues to grow. This stability makes the ONEST method valuable for predicting biomarker performance in large-scale practice. Moreover, the plateau point reveals the minimum number of pathologists necessary to obtain reliable concordance estimates when the assay is widely implemented. The ggplot2 package was used for plotting and data visualizations in this study.

Table S1. Clinicopathological characteristics of patients

Parameter	Total (n=460)
Gender	
Male	316(68.7%)
Female	144(31.3%)
Specimen	
Biopsy	54 (11.7%)
Surgery	406(88.3%)
Site	
Cardia	67 (14.6%)
Gastric body	187(40.7%)
Antrum-pylorus	175(38.0%)
Multiple sites	31 (6.7%)
T stage	
T1	125(27.2%)
T2	50 (10.9%)
T3	67 (14.6%)
T4	125(27.2%)
NA	93 (20.2%)
N stage	
N0	173(37.6%)
N1	56 (12.2%)
N2	61 (13.3%)
N3	77 (16.7%)
NA	93 (20.2%)
Differentiation	
Low	124(27.0%)
Moderate	276(60.0%)
High	60 (13.0%)
Lauren type	
Diffuse	95 (20.7%)
Intestinal	236(51.3%)
Mixed	129(28.0%)
MMR	
pMMR	416(90.4%)
dMMR	44 (9.6%)
EBER	
Positive	16(3.5%)
Negative	399(86.7%)
NA	45 (9.8%)

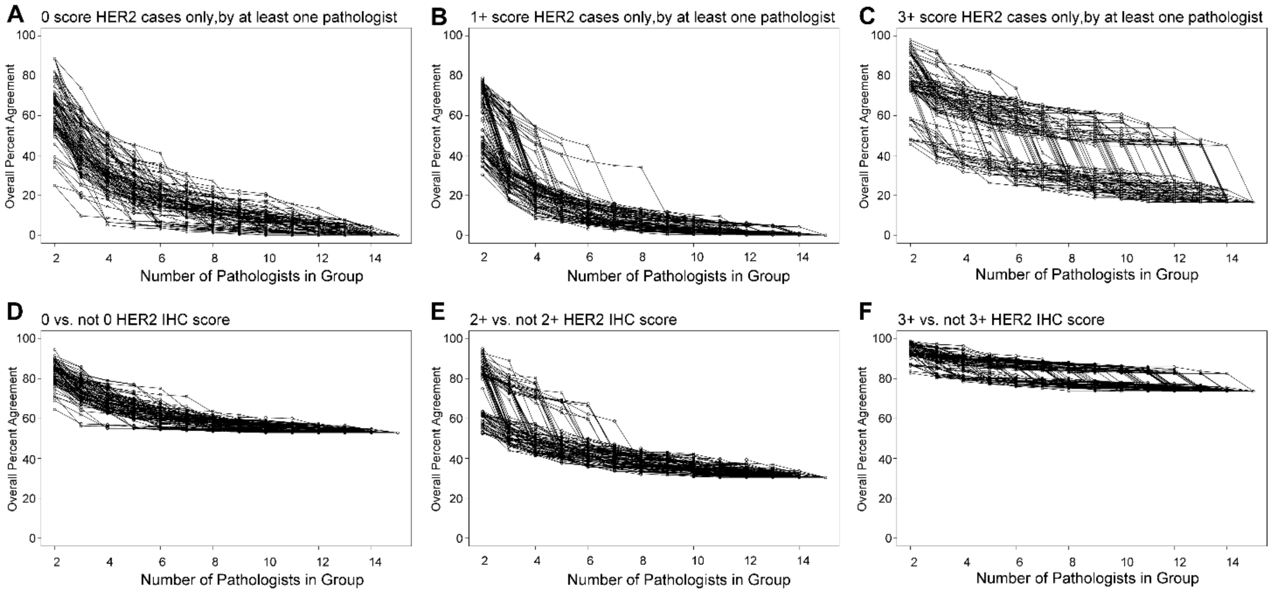

Figure S1. Observers Needed to Evaluate Subjective Tests (ONEST) plots of overall percent agreement (OPA) for different HER2 IHC groupings. OPA ONEST plots for the subset of cases that were read as (A) HER2 IHC 0 or (B) 1+ or 3+ by at least 1 of the 15 pathologist raters. OPA ONEST plots of determining HER2 IHC score of (D) 0 *vs.* not 0, (E) 2+ *vs.* not 2+ or (F) 3+ *vs.* not 3+. HER2, human epidermal growth factor 2; IHC, immunohistochemistry.

Table S2. Summary of interrater reliability metrics for different HER2 IHC groups among pathologists with ≥5 years versus <5 years of experience in interpreting gastrointestinal tumor slides.

	HER2 IHC group	Overall percent agreement(95%)	Fleiss'kappa(95%)	ICC(95%)
	4-category score (0, 1+, 2+, 3+)	0.18(0.15~0.22)	0.44(0.44~0.44)	0.68(0.64~0.71)
	3-category score (0, low[a], 3+)	0.46(0.42~0.51)	0.54(0.54~0.54)	0.74 (0.71~0.77)
	Including cases with only this score by at least 1 pathologist			
	0 only	0(0~0.03)	0.11(0.11~0.11)	0.17(0.12~0.23)
	1+ only	0.02(0.0~0.04)	0.31(0.31~0.31)	0.52(0.48~0.57)
**<5 Years**	2+ only	0.14(0.11~0.19)	0.35(0.35~0.35)	0.49(0.44~0.54)
	3+ only	0.38(0.28~0.49)	0.30(0.30~0.30)	0.48(0.39~0.58)
	low only	0.42(0.38~0.47)	0.37(0.37~0.37)	0.58(0.54~0.62)
	0 vs not 0	0.54(0.49~0.58)	0.45(0.45~0.45)	0.45(0.41~0.49)
	low vs not low	0.46(0.42~0.51)	0.49(0.49~0.49)	0.49 (0.45~0.53)
	3+ vs not 3+	0.89(0.85~0.91)	0.80(0.80~0.80)	0.80 (0.78~0.82)
	<2+ vs ≥2+	0.48(0.43~0.52)	0.53(0.53~0.53)	0.54 (0.50~0.58)
	**HER2 IHC group**	**Overall percent agreement(95%)**	**Fleiss'kappa(95%)**	**ICC(95%)**
	4-category score (0, 1+, 2+, 3+)	0.40(0.35~0.44)	0.56(0.56~0.56)	0.69(0.66~0.72)
	3-category score (0, low[a], 3+)	0.52(0.47~0.56)	0.55(0.55~0.55)	0.61(0.58~0.65)
	Including cases with only this score by at least 1 pathologist			
	0 only	0.07(0.04~0.11)	0.22(0.22~0.22)	0.24(0.18~0.30)
	1+ only	0.00(0.00~0.03)	0.25(0.25~0.25)	0.38 (0.32~0.44)
**≥5 Years**	2+ only	0.43(0.38~0.49)	0.41(0.41~0.41)	0.48 (0.43~0.52)
	3+ only	0.18(0.13~0.26)	0.46(0.46~0.46)	0.59 (0.52~0.66)
	low only	0.49(0.44~0.54)	0.48(0.48~0.48)	0.61 (0.57~0.65)
	0 vs not 0	0.66(0.62~0.70)	0.51(0.51~0.51)	0.51 (0.47~0.55)
	low vs not low	0.54(0.49~0.58)	0.55(0.55~0.55)	0.55 (0.51~0.59)
	3+ vs not 3+	0.77(0.73~0.81)	0.64(0.64~0.64)	0.64 (0.60~0.67)
	<2+ vs ≥2+	0.62(0.57~0.66)	0.64(0.64~0.64)	0.70 (0.67~0.73)

HER2, human epidermal growth factor 2; IHC, immunohistochemistry. ICC, intraclass correlation coefficient a. The low category is the result of combining the 1+ and 2+ categories. 95%CI, 95% Confidence Interval.

Table S3. Summary of interrater reliability metrics among different specimen types.

	HER2 IHC group	Overall percent agreement(95%)	Fleiss'kappa(95%)	ICC(95%)
	4 category (0, 1+, 2+, 3+)	0.22(0.13~0.35)	0.47(0.470~0.47)	0.71(0.62~0.79)
	3 category (0, low[a],3+)	0.28(0.18~0.41)	0.50(0.50~0.50)	0.70(0.62~0.79)
	Including cases with only this score by at least 1 pathologist			
	0 only	0(0~0.10)	0.21(0.21~0.21)	0.28(0.19~0.42)
	1+ only	0(0~0.09)	0.21(0.21~0.21)	0.34(0.24~0.48)
Biopsy Specimen	2+ only	0.15(0.07~0.31)	0.31(0.31~0.31)	0.51(0.39~0.65)
	3+ only	0.21(0.11~0.38)	0.31(0.31~0.31)	0.71(0.62~0.86)
	low only	0.15(0.07~0.28)	0.36(0.36~0.36)	0.55(0.44~0.66)
	0 vs not 0	0.35(0.24~0.49)	0.38(0.38~0.38)	0.39(0.30~0.50)
	low vs not low	0.26(0.16~0.39)	0.42(0.42~0.42)	0.42(0.33~0.54)
	3+ vs not 3+	0.70(0.57~0.81)	0.74(0.74~0.74)	0.75(0.67~0.82)
	<2+ vs ⩾2+	0.44(0.32~0.58)	0.67(0.67~0.67)	0.68(0.59~0.77)
	**HER2 IHC group**	**Overall percent agreement(95%)**	**Fleiss'kappa(95%)**	**ICC(95%)**
	4 category (0, 1+, 2+, 3+)	0.13(0.10~0.16)	0.40(0.40~0.40)	0.60(0.57~0.64)
	3 category (0, low,3+)	0.40(0.36~0.45)	0.49(0.49~,0.49)	0.62(0.59~0.66)
	Including cases with only this score by at least 1 pathologist			
	0 only	0(0~0.02)	0.22(0.22~0.22)	0.27(0.22~0.32)
	1+ only	0(0~0.01)	0.27(0.27~0.27)	0.44(0.40~0.49)
Surgical Specimen	2+ only	0.10(0.08~0.14)	0.29(0.29~0.29)	0.45(0.40~0.49)
	3+ only	0.14(0.09~0.21)	0.43(0.43~0.43)	0.63(0.57~0.70)
	low only	0.38(0.33~0.43)	0.35(0.35~0.35)	0.55(0.51~0.59)
	0 vs not 0	0.55(0.50,0.60)	0.43(0.43~0.43)	0.44(0.40~0.47)
	low vs not low	0.41(0.36~0.45)	0.46(0.46~0.46)	0.46(0.43~0.50)
	3+ vs not 3+	0.74(0.70~0.78)	0.66(0.66~0.66)	0.66(0.62~0.69)
	<2+ vs ⩾2+	0.32(0.28~0.37)	0.42(0.42~0.42)	0.43(0.39~0.47)

HER2, human epidermal growth factor 2; IHC, immunohistochemistry. ICC, intraclass correlation coefficient a. The low category is the result of combining the 1+ and 2+ categories. 95%CI, 95% Confidence Interval.

supplemental references

1. Han, G, Schell, MJ, Reisenbichler, ES, Guo, B & Rimm, DL. Determination of the number of observers needed to evaluate a subjective test and its application in two PD-L1 studies. *Stat Med* **41**, 1361-1375 (2022). https://doi.org:10.1002/sim.9282.
